# Interpreting Cytokinin Action as Anterograde Signaling and Beyond

**DOI:** 10.3389/fpls.2021.641257

**Published:** 2021-03-29

**Authors:** Yoshihisa Ikeda, David Zalabák, Ivona Kubalová, Michaela Králová, Wolfram G. Brenner, Mitsuhiro Aida

**Affiliations:** ^1^Centre of the Region Haná for Biotechnological and Agricultural Research, Czech Advanced Technology and Research Institute, Palacký University, Olomouc, Czechia; ^2^Laboratory of Growth Regulators, Palacky University and Institute of Experimental Botany AS CR, Olomouc, Czechia; ^3^Leibniz Institute of Plant Genetics and Crop Plant Research (IPK), Gatersleben, Germany; ^4^General and Applied Botany, Institute of Biology, Universität Leipzig, Leipzig, Germany; ^5^International Research Organization for Advanced Science and Technology (IROAST), Kumamoto University, Kumamoto, Japan

**Keywords:** anterograde signaling, cytokinin, chloroplast, organelle communication, retrograde signaling, shoot apical meristem, tissue culture, *WUSCHEL*

## Abstract

Among the major phytohormones, the cytokinin exhibits unique features for its ability to positively affect the developmental status of plastids. Even early on in its research, cytokinins were known to promote plastid differentiation and to reduce the loss of chlorophyll in detached leaves. Since the discovery of the components of cytokinin perception and primary signaling, the genes involved in photosynthesis and plastid differentiation have been identified as those directly targeted by type-B response regulators. Furthermore, cytokinins are known to modulate versatile cellular processes such as promoting the division and differentiation of cells and, in concert with auxin, initiating the *de novo* formation of shoot apical meristem (SAM) in tissue cultures. Yet how cytokinins precisely participate in such diverse cellular phenomena, and how the associated cellular processes are coordinated as a whole, remains unclear. A plausible presumption that would account for the coordinated gene expression is the tight and reciprocal communication between the nucleus and plastid. The fact that cytokinins affect plastid developmental status via gene expression in both the nucleus and plastid is interpreted here to suggest that cytokinin functions as an initiator of anterograde (nucleus-to-plastid) signaling. Based on this viewpoint, we first summarize the physiological relevance of cytokinins to the coordination of plastid differentiation with *de novo* shoot organogenesis in tissue culture systems. Next, the role of endogenous cytokinins in influencing plastid differentiation within the SAM of intact plants is discussed. Finally, a presumed plastid-derived signal in response to cytokinins for coupled nuclear gene expression is proposed.

## Introduction

Since their discovery over 60 years ago, cytokinins (CKs), in concert with auxin, have been shown to promote cell division and shoots in an *in vitro* tissue culture system (Miller et al., 1955; Skoog and Miller, 1957), while a treatment of kinetin alone mitigated the chlorophyll losses in detached *Xanthium* leaves (Richmond and Lang, 1957). Although dark-grown seedlings treated with CKs exhibited de-etiolated traits, it appears that CKs and light signaling operate independently or sequentially via partially overlapping pathways (Chory et al., 1994). Myriad studies confirm the positive effects of CKs on plastids’ functioning: protecting them from high light-induced damage, and preventing chlorophyll degradation under dark conditions (Cortleven and Schmülling, 2015). Indeed, the expression of genes encoding a subset of enzymes in the chlorophyll biosynthesis pathway and those encoding the multiple subunits of the light-harvesting complex (LHC) were augmented by a CK treatment (Cortleven et al., 2016).

All plastids present in aboveground tissues are derived from those in the central zone (CZ) of the shoot apical meristem (SAM). The developmental status of plastids in the SAM was considered proplastid (Lopez-Juez and Pyke, 2005; Fleming, 2006; Sakamoto et al., 2008) until recently, when Charuvi et al. (2012) revealed that it varied in the shoot apex, depending on the position or layer they were located: plastids in the CZ and peripheral zone (PZ) of the L1 and L3 could partially develop thylakoid membranes. Several studies demonstrated the CZ of L3 (organizing center/OC; also referred to as the rib meristem) is the site for endogenous cytokinin perception (Gordon et al., 2009; Zürcher et al., 2013; Gruel et al., 2016; Snipes et al., 2018). Despite the fact that plastids in endogenous CK-rich domains are poorly developed, a high-resolution gene expression map in the SAM uncovered greater expression of genes encoding the photosystem I (PSI) LHC A and its subunit proteins, as well as photosystem II (PSII) components and its subunit protein in the L3, by >1.5-fold (relative to other domains) (Yadav et al., 2014). These findings suggest the CZ of the shoot apex provides a unique system for investigating the impact of endogenous CKs in the early phase of plastid development.

**FIGURE 1:**
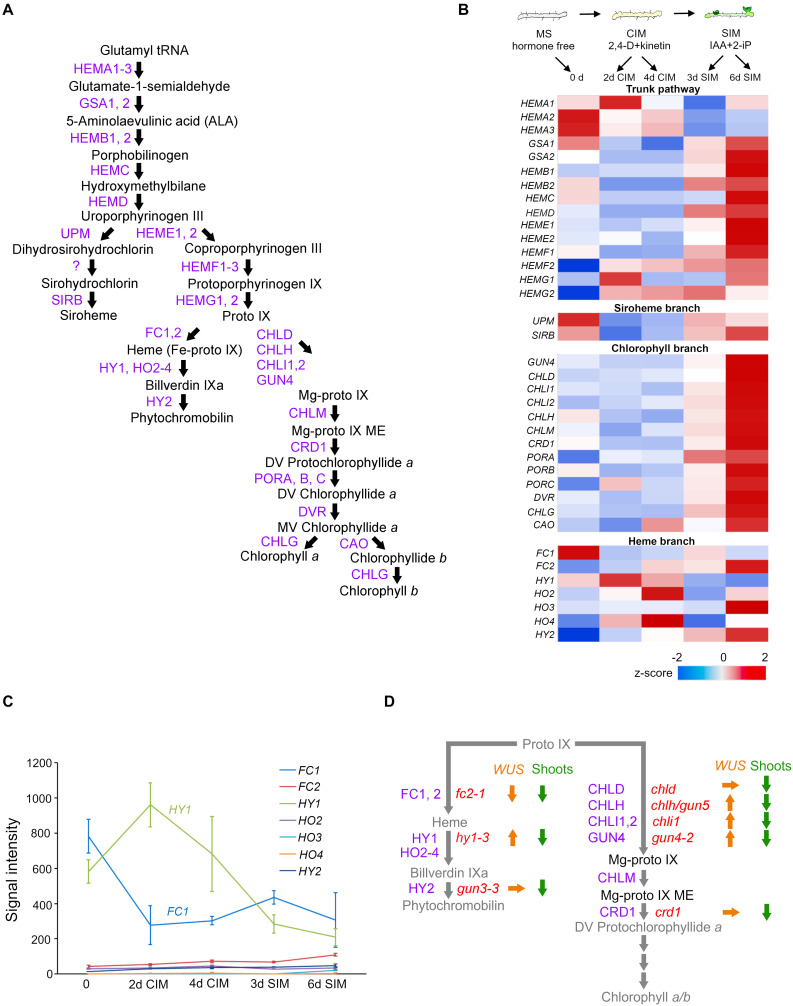
The relationship between the tetrapyrrole biosynthesis pathway and cytokinin-mediated *de novo* shoot apical meristem (SAM) development in tissue culture. **(A)** Tetrapyrrole biosynthesis pathway. Arrows indicate enzymatic reactions. Abbreviations of enzyme names are depicted in purple. **(B)** Schematic morphological representation of excised root explants cultured on MS (Murashige and Skoog medium), CIM (callus-inducing medium), and SIM (shoot-inducing medium) (top), and the expression profiles of genes involved in tetrapyrrole biosynthesis at indicated time points (day) of the indicated culture medium (bottom). Relative expression levels (*z*-scores) are displayed using the color code (blue to red). All the data came from the Arabidopsis eFP browser (http://bar.utoronto.ca/efp_arabidopsis/cgi-bin/efpWeb.cgi?dataSource~=~Regeneration). **(C)** Signal intensity of genes involved in the heme branch. **(D)** Summary of relative expression levels of *WUS* (orange arrows) and shoot regeneration efficiency (green arrows) in root explants of tetrapyrrole mutants cultured on SIM. Up, down, and right arrows indicate an increased *WUS* transcript level, decreased *WUS* transcript level or compromised shoot regeneration, or statistically insignificant changes in the *WUS* transcript level, respectively. The names of enzymes and corresponding mutants are, respectively, depicted in purple and red.

In this perspective article, we attempt to interpret the action of CK as anterograde signaling. As proposed in prior reviews, the term “anterograde signaling” refers to the process whereby nucleus-derived regulators that transmit information express proteins to coordinate the expression of genes in the nucleus and plastids at multiple levels (Pesaresi et al., 2007; Kleine et al., 2009; Jung and Chory, 2010; Berry et al., 2013). In this way, multiprotein complex machineries, such as photosystems, cytochrome b6/f or ATP synthase, are optimally assembled (Kleine et al., 2009). We begin by focusing on how exogenous and endogenous CKs affect the expression of genes encoding tetrapyrrole biosynthesis, a fundamental metabolic pathway in all living organisms (Figure 1A; Tanaka et al., 2011), and then summarizing these effects. Although all genes involved in tetrapyrrole biosynthesis are in the nuclear genome, we scrutinized them because some were found to be CK-responsive (reviewed by Cortleven and Schmülling, 2015). Lastly, based on the results obtained from an *in vitro* tissue culture system (Kubalová et al., 2019), the plausible involvement of retrograde signaling in modulating expression of the nuclear gene *WUSCHEL (WUS)*, a master regulator of stem cell niche, is explored.

## Initiation of Anterograde Regulation by CKs

An exogenous CK treatment can greatly impact plastids’ development and functioning (Cortleven and Schmülling, 2015). Gene expression analyses have corroborated this, by uncovering upregulated plastid-encoded genes upon CK treatment in Arabidopsis and barley (Brenner et al., 2005; Zubo et al., 2008; Danilova et al., 2017a,b; Andreeva et al., 2020). In Arabidopsis, upregulated plastid genes include components of cytochrome b6/f (*petA*) and PSII (*PsbG*, *psbA*, and *psbl*) (Brenner et al., 2005). Both machineries consist of protein complexes encoded in the nucleus and plastids. Additionally, CK indirectly affects plastids’ gene expression by upregulating nuclear genes that encode components of their transcription machinery (i.e., phage-type RNA-polymerases, sigma factors, and plastid RNA polymerase-associated proteins; Danilova et al., 2017a; Andreeva et al., 2020). These results imply that CK-coordinated gene expression activity in the nucleus and plastid provides appropriate information for balancing metabolic flows in plastids and cellular functions.

## Relationship Between Plastid Status in the Sam and CK Signaling in Tissue Culture

The *in vitro* tissue culture technique has been employed to study pluripotency. In such a system, *de novo* organogenesis is manipulated by a defined cytokinin-to-auxin ratio in cultured medium. As Figure 1B (top) illustrates, this widely used method has two sequential steps (Valvekens et al., 1988). The first involves pre-incubation of explants on an auxin-rich callus-inducing medium (CIM), to induce a mass of growing cells, termed a callus. The second step promotes the greening of foci when these induced calli are cultured on shoot-inducing medium (SIM) to which a high cytokinin-to-auxin ratio has been applied. The underlying mechanisms of these processes are detailed in several recent review articles (Shin et al., 2020; Xu and Hu, 2020). The external application of CKs induces *WUS* expression likely via direct activation by type B Arabidopsis Response Regulators (B-ARRs) (Dai et al., 2017; Meng et al., 2017; Zhang et al., 2017; Zubo et al., 2017; Xie et al., 2018); albeit a high CK concentration is required (Gordon et al., 2009). The tight linkage between CK-stimulated *WUS* expression and *de novo* shoot regeneration has been corroborated by genetic studies. Loss-of-function mutations in *WUS* compromised *de novo* shoot regeneration (Gordon et al., 2007; Chatfield et al., 2013; Zhang et al., 2017). Mutants with decreased CK perception were characterized by poor shoot regeneration (Ueguchi et al., 2001; Nishimura et al., 2004; Riefler et al., 2006; Pernisova et al., 2018). By contrast, root explants of CK-hypersensitive mutants—*ckh1* defective in the gene encoding TATA-box binding protein (TBP)-associated factor TAF12-like protein, or *ckh2/pkl (pickle)* defective in gene encoding CHD3 class of SWI/SNF chromatin remodeling factor—promoted tissue greening on CIM culture (Furuta et al., 2011) and developed *de novo* shoots more rapidly and more frequently during the course of SIM incubation. The shoot regeneration efficiencies in the mutants with altered CK perception coincided well with their *WUS* expression levels (Kubalová and Ikeda, 2017; Kubalová et al., 2019).

## Changes to Tetrapyrrole Biosynthesis Gene Expression at the Onset of Shoot Regeneration

As expected from the greening of a tissue phenotype of root explants cultured on SIM (Figure 1B top), analysis of a previously reported microarray expression profile (eFP browser)^1^ (Che et al., 2002) confirms the increased expression of genes involved in the pathway leading to the chlorophyll *a/b* (trunk pathway and chlorophyll branch) (Figure 1B). The exception was the *HEMA* genes, whose products catalyze the initial step in the trunk tetrapyrrole pathway (Kumar and Söll, 2000), their transcripts were substantially reduced when root explants were transferred from CIM onto SIM. This is very intriguing because *HEMA1*, encoding GLUTAMYL-tRNA-REDUCTASE (GluTR), is mainly expressed in photosynthetic tissues, regulated by light through phytochromes (Papenbrock et al., 1999; McCormac et al., 2001), and induced by CK when applied to dark-grown seedlings (Cortleven et al., 2016). In stark contrast to the increased expression of genes involved in the chlorophyll branch, the expression of genes involved in the heme branch did not respond well to SIM incubation. Rather, transcripts of *LONG HYPOCOTYL 1 (HY1)/HEME OXYGENASE 1 (HO1)* declined over time (Figure 1B). The expression levels of *HO3* and *HO4* increased, but their contribution seems minor because the signal intensities of their transcripts are negligible when compared to that of *HY1* (Figure 1C). Although no quantification results of tetrapyrrole molecules during SIM incubation are currently available, we may reasonably speculate that this reduced *HEMA* expression is due to an insufficient availability of phytochromobilin, the chromophore of phytochromes. It has been repeatedly documented that a wide range of phytochrome-mediated light responses become impaired in *hy1* plants; this suggests light-induced *HEMA1* expression is impaired when the *HY1*’s transcription diminished under the SIM incubation. Notably, HEMA enzymatic activity is attenuated by heme accumulation, in a feedback mechanism (Pontoppidan and Kannangara, 1994), and HEMA proteins are destabilized by heme (Richter et al., 2019). Nevertheless, the *HEMA* transcripts in *hy1* are comparable to those in the wild type (Goslings et al., 2004). Collectively, those findings suggest that, although expression of the rate-limiting *HEMA1* is repressed, chlorophyll *a/b* can readily accumulate during SIM incubation (as evinced by tissue greening). Conversely, the heme branch is likely to be attenuated by decreased expression of *HY1*, implying that heme accumulation occurs when *de novo* SAM is being initiated in the CK-enriched culture. Further analysis to determine the tetrapyrrole molecule contents during cytokinin-stimulated *de novo* SAM emergence is required.

## *WUS* Expression Is Uncoupled From *De Novo* Sam Formation in Mutants Defective in Tetrapyrrole Biosynthetic Pathway

Microarray results also validate the greater expression of meristem-related genes upon CK-rich SIM incubation (Che et al., 2002). B-ARRs encoding GARP transcription factors directly activated *WUS* expression (Meng et al., 2017; Zhang et al., 2017; Xie et al., 2018). Loss-of-function mutations of tetrapyrrole biosynthesis provide a valuable tool to study the relationship between tissue greening and shoot regeneration. All the mutants defective in the heme branch (*protoporphyrin ix ferrochelatase 2/fc2*, *hy1*, *genome-uncoupled 3/gun3*), and chlorophyll branch (*gun4*, *Mg-chelatase d/chld*, *chlh*/*gun5*, *chli1*, *copper response deficient 1/crd1*) exhibiting reduced chlorophyll contents resulted in decreased shoot regeneration efficiency (Kubalová et al., 2019); hence, proper chlorophyll contents are required for the acquisition of *de novo* SAM competence or shoot regeneration efficiency. Unexpectedly, of eight tetrapyrrole biosynthesis mutants with substantially compromised shoot regeneration efficiency, in four (*hy1-3*, *gun4-2*, *chlh/gun5*, *chli1*) the expression level of *WUS* significantly increased (Kubalová et al., 2019). This suggests tetrapyrrole intermediates influence nuclear-encoded *WUS* expression. Notably, *WUS* was expressed most in the *hy1-3* mutant despite its shoot regeneration efficiency being among the lowest (Kubalová et al., 2019). In summary, *WUS* expression in *hy1-3*, *gun4-3*, *chlh*, and *chli1* mutants is uncoupled from shoot regeneration efficiency (Figure 1D), suggesting that, in response to the altered plastid developmental status stimulated by the high cytokinin-to-auxin ratio in SIM, plastid-to-nucleus communication occurs to fine-tune nuclear *WUS* expression during *de novo* shoot organogenesis.

## Altered Plastid Differentiation Status in the SAM

All aboveground tissues arise from the SAM, which contains stem cells in its CZ. The plastids in SAM were once considered proplastids (Lopez-Juez and Pyke, 2005; Fleming, 2006; Sakamoto et al., 2008). Despite that prevailing concept, sophisticated morphological analyses of plastids in Arabidopsis SAM revealed their developmental status was not necessarily null and, depending on their position within the SAM, it had dynamic features: plastids underwent different developmental processes that could lead to either the acquisition or loss of thylakoid membranes (Charuvi et al., 2012). The outermost layer L1 (protoderm) gives rise to the epidermis whose photosynthetic activity is lost once leaves differentiate. Yet, plastids within L1 layer’s cells of the SAM (both in CZ and PZ) developed partially differentiated thylakoid networks. The subepidermal layer L2, located below L1, is the progenitor of outer mesophyll tissue. Despite the fact that the L2 is the major source of photosynthetic tissues, the plastids in the CZ of L2 persist in the most undeveloped state, devoid of thylakoid networks characteristic of proplastids. By contrast, plastids in the PZ cells of L2 develop thylakoid membranes. Beneath them is L3, a multilayer where a small group of cells comprise an OC whose cell fate remains undifferentiated. Inner tissues, such as inner mesophyll and vascular cells, originate from the L3. The differentiation status of plastids in L3’s uppermost layer in CZ is similar to that of L2 in CZ (proplastids), whereas plastids in L3’s inner layers are closer to maturity, having partially developed thylakoid networks.

**FIGURE 2:**
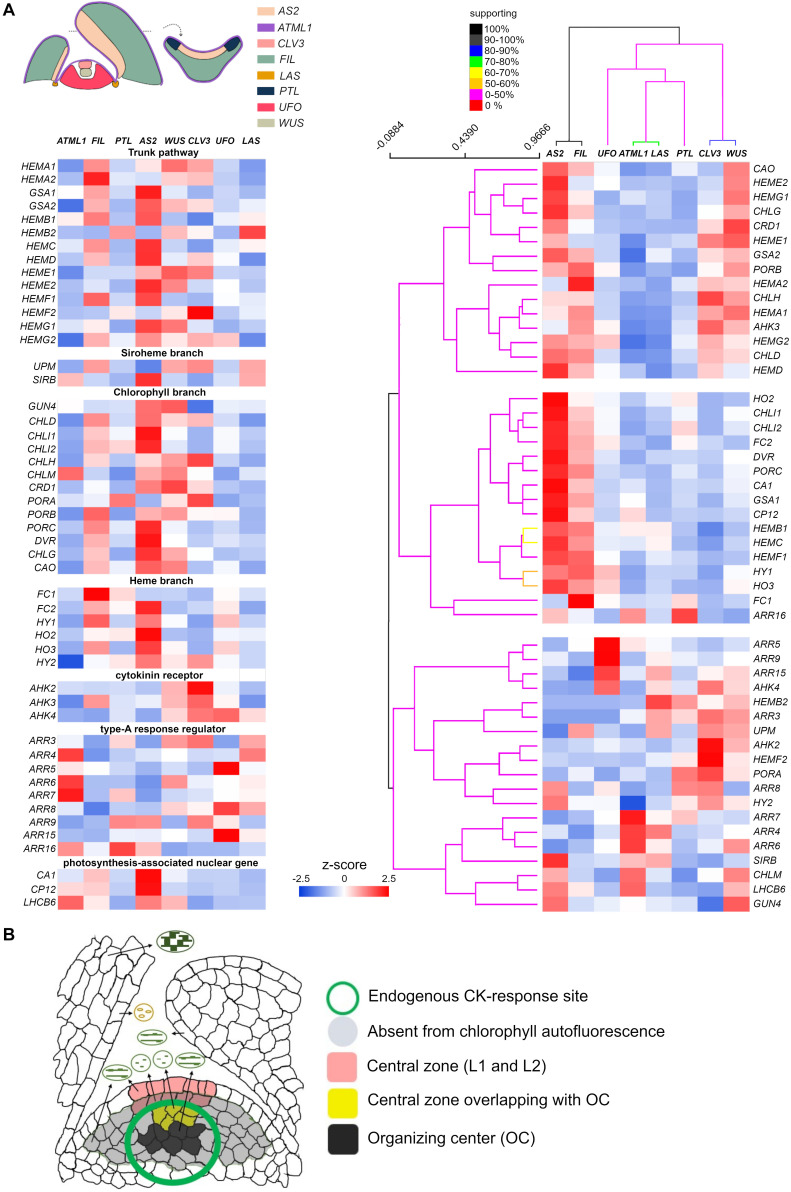
SAM as a unique tissue to study endogenous cytokinin (CK) action on early developmental status of plastids. **(A)** Expression of genes involved in tetrapyrrole biosynthesis, with CK-related and light-responsive genes serving as references for CK or light perception, respectively. The same sets of genes are arranged according to the functional category (left) and to their expression patterns deduced from the clustering analysis (right). Genes and shoot apex domains shown in the left panel were clustered for similarity, in terms of their gene expression pattern, by the Support Tree algorithm implemented in TIGR MeV4 (Saeed et al., 2003), using *n* = 100 iterations of bootstrapping both genes and shoot apex domains. Relative expression levels determined by the *z*-scores are color-coded (in blue to red). Percentage of support for the clustering analysis is depicted according to a color intensity gradient [from red (0%) to black (100%)]. All the data came from the Arabidopsis eFP browser (http://bar.utoronto.ca/efp_arabidopsis/cgi-bin/efpWeb.cgi?dataSource~=~Shoot_Apex), except *HEMA3*, *HO4*, and *ARR17* (Tian et al., 2019). **(B)** Summary of plastid differentiation status in cells differentially positioned in the shoot apex. The plastid status in various types of cells is illustrated, with those cells lacking autofluorescence highlighted in gray [adapted from Charuvi et al., 2012)]. Cells able to respond to endogenous CKs (as revealed by *TCS* reporters) are indicated by a green circle (Zürcher et al., 2013). The L1 and L2 cells within the CZ of SAM are highlighted in orange; the cells in L3 overlapping with the organizing center (OC) appear in yellow. Cells comprising the OC are shown in black.

So the developmental status of plastids within the SAM seems under internal control, and light signaling does not appear to figure prominently because the plastids can better develop thylakoid networks in the inner L3 than in L2. Expression of a CK-reporter gene, *TCS (Two-component Signaling Sensor*) and its derivative *TCSn*, indicated that the primary CK-signaling response was confined within the CZ of L3 (Gordon et al., 2009; Zürcher et al., 2013; Snipes et al., 2018). The majority of molecules participating in primary CK signaling—ARABIDOPSIS HISTIDINE KINASE/AHK receptors, AHP phosphotransmitters, and B-ARRs—are expressed to some extent in Arabidopsis SAM (Nishimura et al., 2004; Gordon et al., 2009; Gruel et al., 2016). Three CK receptors, *AHK2–AHK4*, are predominantly expressed in L3 cells (Gruel et al., 2016) and *WUS*-expressing cells coincide well with *AHK4*-expressing cells (Gordon et al., 2009). Recently, using several tissue-specific promoters, Tian et al. (2019) determined the differential expression pattern of translating RNAs in differing shoot domains, termed “domain-specific translatome map.” To gain insight into CK action’s relation to plastid differentiation status in the SAM, we extracted expression profiles of genes involved in tetrapyrrole biosynthesis, whose results were standardized and conveyed here using *z*-scores (Figure 2A). Although for three CK receptor genes, their expression is reportedly absent in L1 (Gruel et al., 2016), their abundant expression in domains of *CLV3* (*CLAVATA 3*) and *WUS* agree well with those of high-CK responses revealed by expression of the *TCS* reporters (Figure 2A). These results strongly suggest the SAM, especially the CZ of L3, is the active site for the perception of endogenous CKs. Nevertheless, those plastids within L2 and L3 remained the least differentiated; in fact, chlorophyll autofluorescence in the corresponding region was below the detection threshold (Figure 2B; Charuvi et al., 2012; Yadav et al., 2019). Unlike for poorly developed plastid conditions in the *CLV3* or *WUS* domain, several tetrapyrrole biosynthesis genes (*HEMA1*, *HEME1*, *HEMF2*, *CHLH*, *CRD1*, and *PORA*) exhibited the highest transcript levels (Figure 2A), notwithstanding the fact that the expression of *HEMA1*, *CHLH*, and *CRD1* was predominantly induced by light in photosynthetic tissues (Matsumoto et al., 2004). These results suggest that a handful of genes involved in tetrapyrrole biosynthesis are not only regulated by light but also by intrinsic signal(s). A plausible candidate for this might be CKs or the interplay between them and other hormones. It appears that upregulation of the above genes contributes little or negligibly to chlorophyll biosynthesis in the CZ of L2 and L3, since chlorophyll autofluorescence is undetectable in these layers (Figure 2B; Charuvi et al., 2012; Yadav et al., 2019). The expression of genes involved in the heme branch is generally low in both *CLV3* and *WUS* domains, especially that of *HY1* (lowest in the *CLV3* domain; Figure 2A), indicative of heme accumulation. To gain further insight into the negative relationship between *HY1*’s expression and CK signaling, those genes involved in CKs’ perception and response, as well as light-responsive genes, were analyzed by clustering. These results confirmed that *HY1* fell into a cluster distinct from that harboring the two CK receptors (*AHK2* and *AKH4*) and eight of nine A-ARRs (Figure 2A, right panel). Studying the developmental status of plastids in cells in the CZ of L2 and the uppermost layers of L3 in the CZ, by manipulating endogenous CK levels, would provide a new opportunity to reveal as-of-yet-unknown CK functions in early plastid development. In two different systems, for *de novo* organogenesis in tissue culture and the shoot apices of light-grown seedlings, the *HY1* expression levels are negatively correlated with exogenous CK or endogenous CK-rich domains (Figures 1, 2A). Although further analyses are required to uncover a negative correlation between the two mentioned above, it is likely that, in response to CK as an initiator of anterograde signaling, the plastid-to-nucleus communication happens predominantly in meristematic cells where endogenous CK maxima are established.

## Conclusion and Perspective

Bioactive CKs are molecules perceived by the CHASE domain of CK receptors, for which the primary two-component multistep phosphorelay represents the output module able to regulate gene expression. Given the fact that exogenous CK application to dark-grown plants promotes etioplast-to-chloroplast transition, probably via altered gene expression both in the nucleus and plastids, CKs could positively regulate anterograde signaling to promote plastid differentiation. It appears that the particular developmental and differentiation status affects the sensitivity to CK in plant cells. We propose the CZ of SAM as a promising site to investigate endogenous CK action influencing early plastid development (Figure 2B). An alternative motive for this approach is to gain better insight into the coordinated regulation of stem cell determination vis-à-vis the plastids’ developmental status. The molecular regulatory mechanism underlying diminished expression of *HY1* by CK is not entirely clear. The lower auxin-to-cytokinin ratio in SIM may be responsible for declines in *HY1* expression because *HY1* is induced by auxin and acts downstream of it (Ma et al., 2014). Besides, an auxin minimum is known to exist in the OC of SAM (Shi et al., 2018). Either way, genetic studies have revealed the overlooked contribution of the heme branch (Kubalová et al., 2019). Although tetrapyrrole biosynthesis is controlled under complex regulation, it is reasonable to argue that substrates accumulate when the catalytic enzymatic step is blocked. Since *WUS* expression in *fc2-1* is evidently opposite to that in *hy1-3*, *gun4-3*, *chlh*, and *chli1* (Figure 1D), it is tempting to speculate that heme, whose synthesis is blocked in *fc2-1* but which accumulates in *hy1-3*, is the plausible candidate molecule modulating *WUS* expression. It goes without saying that the alternative interpretations remain open to account for this. Interestingly, *hy1* mutant seedlings accumulate substantially elevated level of endogenous jasmonates by unknown mechanisms and, as a result, jasmonic acid responses are constitutively active (Zhai et al., 2007). Alternatively, the decreased carbon monoxide, a byproduct of heme oxygenase enzymatic reaction, may come into play (Xuan et al., 2008; Ma et al., 2014).

Beyond its diverse functions to serve as cofactors of hemoproteins in various organelles (Layer et al., 2010), several recent studies pose heme as a retrograde signal that is involved in various physiological regulations (Mense and Zhang, 2006; Von Gromoff et al., 2008). Besides, biochemical evidence corroborates this, i.e., half of the identified heme-binding proteins localize in the nucleus in plants (Shimizu et al., 2020). Measuring or monitoring heme content during *de novo* organogenesis and in the CZ of the SAM of intact plants will provide us with a new insight into the role of the heme branch in nuclear gene expression.

## Data Availability Statement

Publicly available datasets were analyzed in this study. This data can be found here: http://bar.utoronto.ca/efp_arabidopsis/cgi-bin/efpWeb.cgi?dataSource=Regeneration; http://bar.utoronto.ca/efp_arabidopsis/cgi-bin/efpWeb.cgi?dataSource=Shoot_Apex.

## Author Contributions

YI conceived the study’s idea. YI, IK, MK, DZ, WB, and MA wrote the manuscript. WB conducted the clustering analysis. All authors contributed to the article and approved the submitted version.

## Conflict of Interest

The authors declare that the research was conducted in the absence of any commercial or financial relationships that could be construed as a potential conflict of interest.

## Footnotes

1 http://bar.utoronto.ca/efp_arabidopsis/cgi-bin/efpWeb.cgi?dataSource=Regeneration
